# fMRI data from Korean, Chinese and English subjects in a word rhyming judgment task

**DOI:** 10.1016/j.dib.2016.03.006

**Published:** 2016-03-09

**Authors:** Fan Cao

**Affiliations:** Department of Communicative Sciences and Disorders, Michigan State University, USA

**Keywords:** fMRI, Reading, Rhyming, Chinese, Korean, English

## Abstract

This article includes the description of data information from a visual word rhyming judgment task in native Korean, native Chinese and native English speakers. You will find fMRI data information including experimental design, MRI protocol, and brain activation results from a conjunction analysis of the three groups of subjects. Other results from the same study were published in “How does language distance between L1 and L2 affect the L2 brain network? An fMRI study of Korean–Chinese–English trilinguals” (Kim et al., 2015 [1]).

**Specifications table**

**Table:** 

Subject area	*Psychology*
More specific subject area	*fMRI of reading*
Type of data	*Table*
How data was acquired	*fMRI*
Data format	*Analyzed*
Experimental factors	*Language: Korean, Chinese and English*
Experimental features	*Visual word pairs were sequentially presented, and subjects were told to press a button if they rhyme and another button if they do not rhyme*
Data source location	*East Lansing, Michigan, USA*
Data accessibility	*Data is within this article*

## Value of the data

• Current data can be used to examine the universal brain activation during single word reading across different languages.
• Current data can be used to compare to monolingual speakers brain activation.
• Current data can be used to examine higher cognitive function in the adults’ brain such as reading and phonological processing.

## 1 Data

The fMRI data comes from a word rhyming judgment task in native Korean speaker, native Chinese speakers and native English speakers [1]. The fMRI data were the result of the conjunction brain activation in the three groups of subjects. In other words, the brain activation reported in this article is the overlapped activation in the three languages.

## 2 Experimental design, materials and methods

### 2.1 Task

During functional magnetic resonance imaging (fMRI), participants performed a rhyming judgment task on sequentially presented visual word pairs in one of the three languages (Korean, Chinese, or English), mixed with perceptual control and baseline trials. Participants were instructed that they would see word pairs on the screen one at a time and should decide as quickly and as accurately as possible whether the two words rhymed or not, using their right index finger for “yes” and their right middle finger for “no.” For each trial, each stimulus was presented for 800 ms, with a 200 ms blank interval between the stimuli. A red fixation cross appeared on the screen immediately after the offset of the second stimulus in the stimuli pair, indicating the need to make a response. The response interval duration varied (2200, 2600 or 3000 ms), such that each trial lasted for either 4000, 4400, or 4800 ms. For resting baseline trials (*N*=48), the participant was required to press the “yes” button when a black fixation cross was presented at the center of the screen. Perceptual control trials (*N*=24) were also included as part of a larger study, and were not of interest in the present experiment. During these trials, participants were required to determine whether two sequentially presented symbol patterns were matched or mismatched by pressing the “yes” or “no” buttons. The timing for the perceptual control and resting baseline trials was the same as for the lexical trials. The order of presentation of lexical, perceptual, and resting baseline trials and the variation of the response interval were optimized for event-related designs by OptSeq [http://surfer.nmr.mgh.harvard.edu/optseq].

For the English and Chinese rhyming judgment tasks, there were 24 trials per condition including two rhyming and two non-rhyming conditions. The two rhyming conditions included one with similar orthographic and phonological endings (O+P+) and one with different orthographic but similar phonological endings (O−P+). The two non-rhyming conditions included one with similar orthographic but different phonological endings (O+P−) and one with different orthographic and phonological endings (O−P−). All English words were monosyllabic without homophones and they were matched across conditions for written word frequency and the sum of their written bigram frequency [English Lexicon Project, http://elexicon.wustl.edu]. All Chinese words consisted of two characters and did not have homophones at the word level. Similar orthography was defined as the same phonetic radical for the second character of the word. In half of the trials of the four lexical conditions (rhyming and non-rhyming), the second character of the words had the same tone (e.g., 弥补 /mi2bu3/, 纯朴/chun2pu3/), and in the other half, they had different tones (e.g., 逮捕/dai4bu3/, 胸脯/xiong1pu2/). The two-character words and the second character of those words were matched across conditions on adult written frequency [2] and number of strokes across conditions.

For the Korean task, there were 24 trials in each of three conditions, two rhyming and one non-rhyming, namely O+P+, O−P+ and O−P−. The O+P− condition is not possible for Korean due to its transparent writing system. As in other language tasks, both resting baseline trials (*N*=24) and perceptual trials (*N*=24) were included. All Korean words were disyllabic without homographs or homophones at the word level. The written frequency of the words was matched across conditions based on the Korean Word Database, Sejong corpus [2003]. In addition, word frequency in all three languages was matched [*F* (2, 516)=2.158, *P*=.117].

### 2.2 MRI data acquisition

All images were acquired using a 3.0 T Siemens scanner (Siemens Healthcare, Erlangen, Germany) at Beijing Normal University. Participants lay in the scanner with their head position secured with foam padding. An optical response box was placed in each participant׳s dominant right hand and a compression alarm ball in the left hand. The head coil was positioned over each participant׳s head in a way that they could effectively use the mirror to view the projection screen at the rear of the scanner. Gradient echo localizer images were acquired to determine the placement of the functional slices. For the functional images, a susceptibility weighted single-shot echo planar imaging (EPI) method with blood oxygenation level-dependency (BOLD) was used with the following scan parameters: TR=2000 ms, TE=20 ms, flip angle=80°, matrix size=120×128, field of view=220×206.3 mm^2^, slice thickness=3 mm (0.48 mm gap), number of slices=32. These parameters resulted in a 1.7×1.7×3 mm voxel size. Using an interleaved bottom-to-top sequence, 145 whole-brain volumes were acquired for each run. A high resolution, T1 weighted 3D image was also acquired using MP RAGE with the following parameters: TR=2300 ms, TE=3.36 ms, flip angle=9°, matrix size=256×256, field of view=256 mm, slice thickness=1 mm, number of slices=160, resulting voxel size=1×1×1 mm^2^. The acquisition of the anatomical scan took approximately 9 min and the fMRI scan for each run was 6 min and 44 s for the Chinese and English task and 4 min and 58 s for the Korean task. There were 2 runs for each language task.

### 2.3 Image analysis

Data analysis was performed by DPARSF (http://rmfri.org/DPARSF) [3] and SPM8 [Statistical Parametric Mapping; http://www.fil.ion.ucl.ac.uk/spm]. The following steps were used for data preprocessing: (1) Slice timing correction for interleaved acquisition using sinc interpolation, (2) 4th degree b-splice interpolation for realignment to the first volume, (3) Trilinear coregistration with the anatomical image, (4) Segmentation of the anatomical image, (5) Normalization of all brains to the standard T1 Montreal Neurological Institute (MNI) adult template with a voxel size=2×2×2 mm^3^ (12 linear affine parameters for brain size and position, 8 non-linear iterations and non-linear basis functions), and (6) 4×4×8 mm^3^ full width half maximum Gaussian kernel smoothing. Up to one volume, where movement exceeded 3 mm in any of the *x*, *y* or *z* dimensions, was replaced with the mean of the images immediately before and after the outlying volume. Participants with >1 volume or >3 mm of movement were excluded from the study. Statistical analyses at the first level were conducted using an event-related design with lexical conditions, perceptual control trials, and baseline trials. A high pass filter with a cutoff period of 128 s was applied. Trials were modeled using a canonical hemodynamic response function (HRF). Conjunction analysis was done using SPM8 to examine the universal brain activation in the three groups of subjects (Table 1).

## Figures and Tables

**Table 1:** Brain Activation for the conjunction analysis in native Korean speaker, native Chinese speakers and native English speakers during the visual word rhyming judgment task.

Anatomical region	H	BA	Voxels	*x*	*y*	*z*	*Z*
Inferior occipital gyrus, middle occipital gyrus, inferior temporal gyrus	L	17,18,19	2349	−44	−66	−14	7.07
Medial frontal gyrus	L	6	357	−4	14	52	6.12
Middle frontal gyrus, inferior frontal gyrus	L	46,9	1370	−48	32	20	5.66
Superior frontal gyrus	L	6	154	−22	−6	54	4.85
Inferior occipital gyrus	R	17,18,19	396	44	−80	−6	4.77
Putamen	L	–	35	−18	10	0	4.28
Lingual gyrus	R	17	57	20	−94	−8	4.19
Cuneus	R	18	60	14	−72	12	4.19
Cuneus	L	18	197	−12	−76	6	4.16
Inferior parietal lobule	L	40	220	−40	−44	44	4.04
Middle occipital gyrus	R	19	21	32	−78	14	3.57
