# Supplementary material for: fMRI data from Korean, Chinese and English subjects in a word rhyming judgment task
Source: Data Brief. 2016 Mar 9;7:591–4. doi: 10.1016/j.dib.2016.03.006 (PMC4802529; doi:10.1016/j.dib.2016.03.006)
Supplement: Supplementary file 1 — Supplementary material [file mmc1.docx]

AUTHOR DECLARATION

I wish to confirm that there are no known conflicts of interest associated with this publication and there has been no significant financial support for this work that could have influenced its outcome.

I confirm that we have given due consideration to the protection of intellectual property associated with this work and that there are no impediments to publication, including the timing of publication, with respect to intellectual property. In so doing I confirm that I have followed the regulations of our institutions concerning intellectual property.

I further confirm that any aspect of the work covered in this manuscript that has involved human patients has been conducted with the ethical approval of all relevant bodies and that such approvals are acknowledged within the manuscript.

Signed by the author as follows:

Fan Cao
